# A Disintegrin and Metalloenzyme (ADAM) 17 Activation Is Regulated by α5β1 Integrin in Kidney Mesangial Cells

**DOI:** 10.1371/journal.pone.0033350

**Published:** 2012-03-08

**Authors:** Pal Gooz, Yujing Dang, Shigeki Higashiyama, Waleed O. Twal, Courtney J. Haycraft, Monika Gooz

**Affiliations:** 1 Department of Medicine, Medical University of South Carolina, Charleston, South Carolina, United States of America; 2 Department of Biochemistry and Molecular Genetics, Center for Regenerative Medicine CEREM, Ehime University Graduate School of Medicine, Ehime, Japan; 3 Department of Regenerative Medicine and Cell Biology, Medical University of South Carolina, Charleston, South Carolina, United States of America; 4 College of Dental Medicine, Craniofacial Biology, Medical University of South Carolina, Charleston, South Carolina, United States of America; National Center for Scientific Research Demokritos, Greece

## Abstract

**Background:**

The disintegrin and metalloenzyme ADAM17 participates in numerous inflammatory and proliferative diseases, and its pathophysiological role was implicated in kidney fibrosis, polycystic kidney disease and other chronic kidney diseases. At present, we have little understanding how the enzyme activity is regulated. In this study we wanted to characterize the role of α5β1 integrin in ADAM17 activity regulation during G protein-coupled receptor (GPCR) stimulation.

**Methodology/Principal Findings:**

We showed previously that the profibrotic GPCR agonist serotonin (5-HT) induced kidney mesangial cell proliferation through ADAM17 activation and heparin-binding epidermal growth factor (HB-EGF) shedding. In the present studies we observed that in unstimulated mesangial cell lysates α5β1 integrin co-precipitated with ADAM17 and that 5-HT treatment of the cells induced dissociation of α5β1 integrin from ADAM17. Using fluorescence immunostaining and *in situ* proximity ligation assay, we identified the perinuclear region as the localization of the ADAM17/α5β1 integrin interaction. In cell-free assays, we showed that purified α5β1 integrin and β1 integrin dose-dependently bound to and inhibited activity of recombinant ADAM17. We provided evidence that the conformation of the integrin determines its ADAM17-binding ability. To study the effect of β1 integrin on ADAM17 sheddase activity, we employed alkaline phosphatase-tagged HB-EGF. Overexpression of β1 integrin lead to complete inhibition of 5-HT-induced HB-EGF shedding and silencing β1 integrin by siRNA significantly increased mesangial cells ADAM17 responsiveness to 5-HT.

**Conclusions/Significance:**

Our data show for the first time that β1 integrin has an important physiological role in ADAM17 activity regulation. We suggest that regulating α5β1 integrin binding to ADAM17 could be an attractive therapeutic target in chronic kidney diseases.

## Introduction

ADAM17, one of the most intensively studied member of the disintegrin and metalloenzyme family was identified as the protease which cleaves the cell-surface-bound form of the inflammatory cytokine, pro-tumor necrosis factor (TNF)α [1], [2]. Since then, a wealth of information has been gathered about the enzyme function: ADAM17 is indispensable to normal development [3], [4], and it has a fundamental role in inflammation through shedding of TNFα [1], [2] and its receptors, TNFRI and II [5], and processing cell adhesion molecules which initiate leukocyte transmigration through the vascular wall [6]. ADAM17 also has an important role in cell-fate decisions by shedding the ectodomain of Notch [7]. ADAM17 participates in protein ectodomain shedding of epidermal growth factor (EGF) receptor ligands such as heparin-binding EGF (HB-EGF), transforming growth factor (TGF)α, and amphiregulin which, in turn, activate the EGF receptor (EGFR) and initiate downstream signaling events leading to cell proliferation, migration, or apoptosis (see recent review [8]). The role of ADAM17 in the so-called “triple membrane spanning signaling” (TMSS) was also investigated: agonists of G protein-coupled receptors (GPCRs) were shown to trans-activate receptor tyrosine kinases (RTK), including the EGFR, by activating ADAM17 and leading to growth factor shedding (for review see [9]). Our group showed previously that bradykinin changes glomerular permeability through ADAM17 activation-induced epiregulin shedding and EGFR transactivation [10]. We also showed that the pro-fibrotic GPCR agonist serotonin (5-HT) induces proliferation of kidney mesangial cells through EGFR transactivation. We identified ADAM17 as the sheddase enzyme that processes the EGFR ligand HB-EGF during 5-HT-induced TMSS in mesangial cells [11]. Further, we also showed that ADAM17 activity is necessary for VEGF-induced MMP-2 activation and angiogenesis [12]. All of the above described processes: inflammation, apoptosis, cell proliferation, and angiogenesis have important pathophysiological role in the development of chronic kidney diseases [13], [14], [15], [16]. Since ADAM17 has a central role in orchestrating these signaling processes, it is crucial to understand how ADAM17 activity is regulated.

Tissue inhibitor of metalloproteinase (TIMP)-3 was identified by current studies as an important negative regulator of ADAM17 activity. Loss of TIMP-3 in mice increased ADAM17 activity and augmented development of interstitial nephrosis and nephritis after unilateral ureteral obstruction [17]. Another interesting study showed that TIMP-3 knockout animals developed hepatic steatosis and adipose tissue inflammation possibly through ADAM17 upregulation [18]. Higher ADAM17 activity also contributed to high fat diet-induced glucose intolerance and insulin resistance in the same animal model [19].

ADAM17 has a multidomain structure: other than a prodomain, it has a catalytic domain, a disintegrin domain, a transmembrane domain, and a cytoplasmic domain. It was shown previously that phosphorylation of the cytoplasmic domain can regulate the enzyme activity; however, these reports are somewhat controversial [20], [21]. The disintegrin domain of the ADAM enzymes is related to snake venom disintegrins and based on their structural similarities it was implicated in binding the cell adhesion receptors integrins. So far, several ADAMs were shown to bind integrins: the recombinant disintegrin fragment of ADAM15 promoted attachment of cells expressing αvβ3 and α5β1integrins, both in an Arg-Gly-Asp-(RGD)-dependent and independent manner, and α9β1 integrin was shown to bind ADAM1, 2, 3, 7, 9, 12, 15, 28, 33 (detailed review on ADAM-integrin binding [22]). Further, the recombinant ectodomain fragment of ADAM17 was shown to mediate cellular adhesion of α5β1 integrin-expressing cells on a plastic surface and to bind purified α5β1 integrin [23]. Also, cellular ADAM17 was shown to co-localize with overexpressed α5β1 integrin in migrating HeLa cells [23]. In these studies, the ADAM-integrin interaction was chiefly explored in the context of cell adhesion, and ADAMs were often regarded as integrin counter-receptors. At this time, we have no data on how integrin binding affects the enzymatic activity of ADAMs.

We showed recently, that ADAM17 co-precipitated with α5β1 integrin in gastric epithelial carcinoma cells [24]. However, we had no data regarding the exact function of the ADAM17/integrin binding, especially whether ADAM17 binding to α5β1 integrin (or any other integrin) regulated the enzyme activity. Moreover, we were uncertain whether a physiological stimulus such as GPCR activation, which results in subsequent activation of ADAM17, affects ADAM17 integrin binding.

In this study, we provide evidence that α5β1 integrin binds to ADAM17 in resting mesangial cells using co-immunoprecipitation and fluorescence co-localization experiments. We quantify the extent of endogenous protein interaction and confirm the location of the ADAM/integrin interaction by fluorescence *in situ* proximity ligation assay. Furthermore, we demonstrate - for the first time - that α5β1 integrin-binding regulates ADAM17 enzymatic activity both in a cell-free system and in mesangial cells *in vivo*. We show that GPCR stimulation leads to ADAM17/α5β1 integrin complex dissociation, which releases the enzyme activity and contributes to HB-EGF shedding in mesangial cells. Our data suggest that regulating α5β1 integrin binding to ADAM17 could be an attractive therapeutic target in chronic kidney diseases.

## Results

### ADAM17 binds to α5β1 integrin in mesangial cells and GPCR stimulation decreases ADAM17 binding to α5β1 integrin

**Figure 1:**
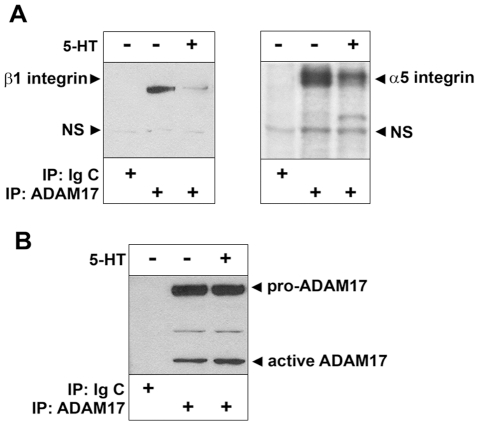
ADAM17 co-precipitates with α5β1 integrin and dissociates from the integrin during GPCR stimulation. (A) Control (−) and 1 µM 5-HT stimulated (+) mesangial cell lysates were immunoprecipitated (IP) with either ADAM17 antibody or Ig control (Ig C), resolved on 3–8% Tris-acetate gel and probed for the presence of β1 integrin and α5 integrin by Western blotting. (B) ADAM17 blot shows that equal amount of ADAM17 were precipitated from each sample. Arrows point to specific and non-specific (NS) bands. One representative example out of four experiments is shown.

To test whether ADAM17 binds to α5β1 integrin in rat kidney mesangial cells we performed immunoprecipitation. Control and 5-HT-treated (1 µM for 10 min) mesangial cell lysates were incubated with ADAM17 antibody. Immunoprecipitated complexes were analyzed by SDS-PAGE followed by Western blotting to visualize the presence of integrins. We found that α5β1 integrin co-precipitated with ADAM17 (Figure 1A). Interestingly, during our experiment we also observed that significantly less integrin co-precipitated with ADAM17 in 5-HT-treated cell lysate than in control unstimulated cells lysate (Figure 1A) suggesting that 5-HT induced dissociation of the ADAM17/α5β1 integrin complex. As negative control we used normal mouse IgG (Figure 1A) and equal loading of immunoprecipitated ADAM17 was confirmed by re-probing the β1 integrin blot for ADAM17 (Figure 1B).

### ADAM17 co-localizes with α5β1 integrin in mesangial cells

**Figure 2:**
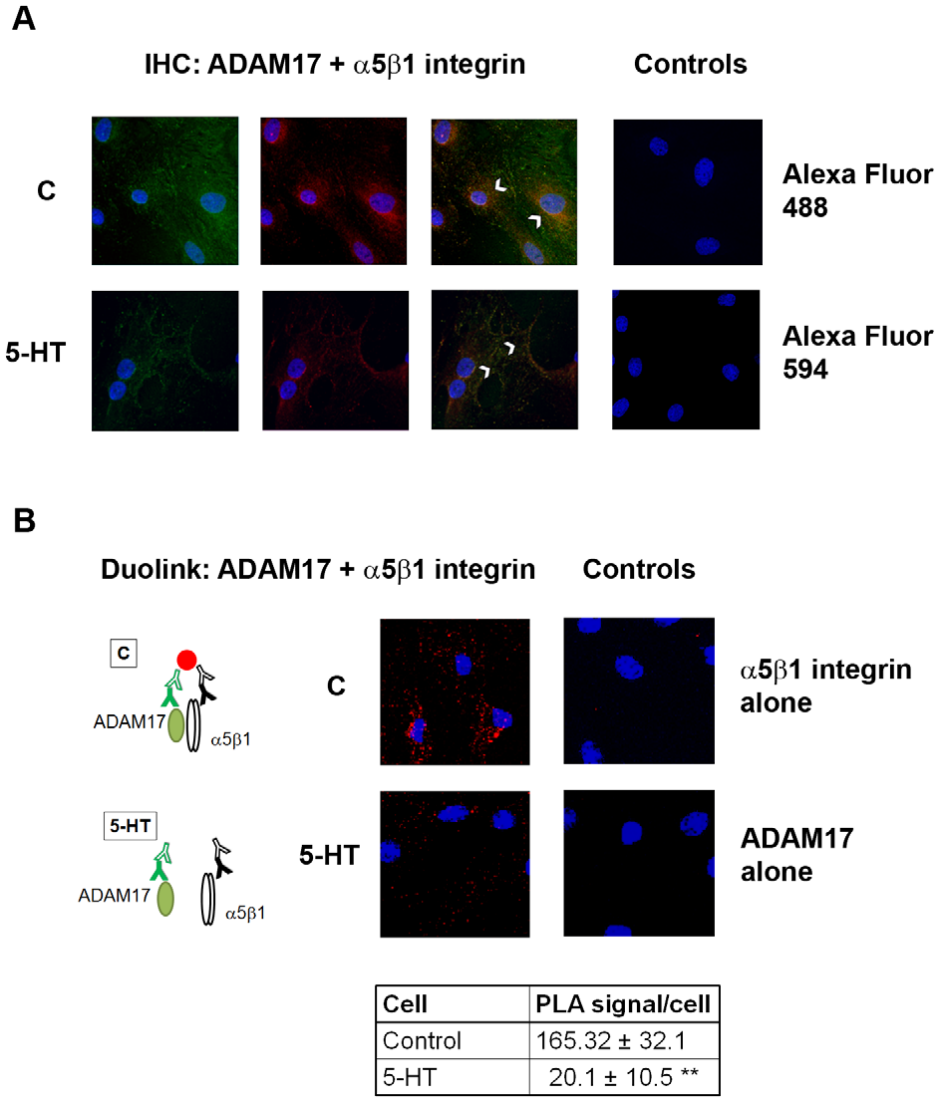
Co-localization of ADAM17 and α5β1 integrin in rat mesangial cells. Control (C) and 1 µM 5-HT -stimulated mesangial cell were fixed, permeabilized, and (A) co-immunostained using ADAM17 antibody (green) and β1 integrin antibody (red) as indicated in “Material and Methods”. Arrows indicate co-localization of ADAM17 and α5β1 integrin immunopositive areas (yellow). For the negative controls we omitted the primary antibodies and used PBS followed by secondary antibodies. (B) Parallel samples were incubated with oligonucleotide-labeled PLA probes after incubation with primary antibodies. PLA signals as fluorescence dots were imaged and quantified. As negative control we used either ADAM17 or α5β1 integrin antibody alone followed by the oligonucleotide-labeled PLA probes. Cartoon explains binding of the fluorescence detection reagent only to antibodies in close proximity; **p<0.01. Representative examples out of three experiments are shown.

To understand the localization of ADAM17/α5β1 integrin interaction we performed immunofluorescence studies. First, we tested the ADAM17 and α5β1 integrin antibodies by a “traditional” fluorescence immunocytochemistry. Quiescent cells were incubated in serum-free medium with or without 1 µM of 5-HT and immunostaining for ADAM17 and α5β1 integrin were performed as detailed in “Material and Methods”. We found that ADAM17 co-localized with the integrin in the perinuclear region of unstimulated cells (Figure 2A upper panels, arrows). After 5-HT stimulation the intensity of both ADAM17 and α5β1 integrin immunopositive signals were decreased in the perinuclear region and rather distributed across throughout the cytoplasm and in the plasma membrane (Figure 2A lower panels, arrows).

Next, we employed the Duolink *in situ* proximity ligation assay (PLA) in order to confirm the co-localization and to quantify the ADAM17/α5β1 integrin interaction. The results obtained with PLA corresponded very well with the results of the immunofluorescence staining. The signals were localized in the perinuclear area in control cells and were disseminated in the cytoplasm in 5-HT-treated cells. Since PLA signal only forms if the two different antibodies (ADAM17 and α5β1 integrin antibodies) bind to proteins that are in close proximity (Figure 2, cartoon), this assay confirmed that ADAM17 forms a complex with α5β1 integrin, and that this complex disassembles upon 5-HT stimulation. Quantitative analysis showed that compared to control cells about 13% of the complexes remained after 5-HT stimulation (Figure 2B).

### Experiments in cell-free systems show direct interaction of recombinant ADAM17 with α5β1 integrin

**Figure 3:**
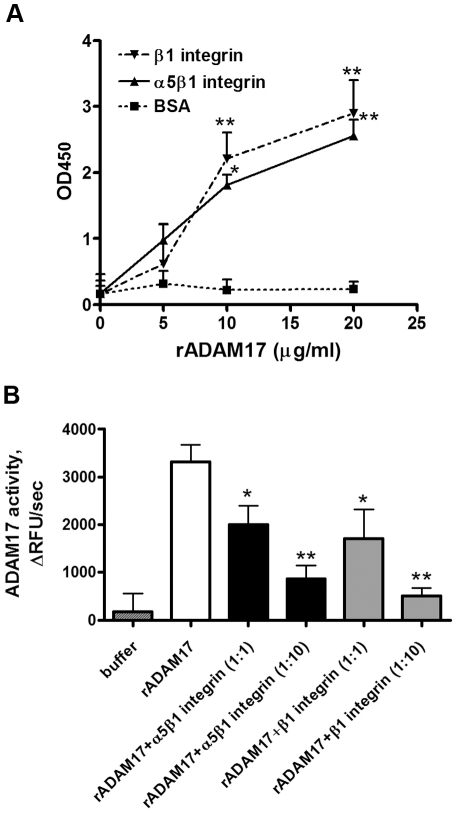
Cell free assays show ADAM17 binding to α5β1 integrin and changes in ADAM17 activity. (A) Recombinant ADAM17 binds purified α5β1 integrin and recombinant β1 integrin in a cell free binding assay. Plates pre-coated with antibodies against α5β1 integrin or β1 integrin were incubated with purified α5β1 integrin or with β1 recombinant integrin, individually. Recombinant ADAM17 was then added at the indicated concentrations and ADAM17 binding was measured using a colorimetric assay at 450 nm as described in Methods. Data are expressed as mean±S.D. *p<0.05, **p<0.01 *vs* control; data from 4 experiments with 3 parallels/each condition are shown. (B) Purified α5β1 integrin and recombinant β1 integrin decrease ADAM17 enzymatic activity. Recombinant ADAM17 (10 ng/ml) was incubated alone or together with either purified α5β1 integrin (25 ng/ml or 250 ng/ml) or with β1 recombinant integrin (12 ng/ml or 120 ng/ml), in OG buffer in the presence of a quenched fluorogenic ADAM17 substrate. Enzyme activity was expressed as the rate of change of relative fluorescence units (ΔRFUs^−1^); *p<0.05, **p<0.01 *vs* control; data from four experiments with eight parallels/each condition are shown.

To investigate whether ADAM17 can bind α5β1 integrin *directly*, even without the cellular environment and/or help of adaptor molecules, we performed the following assays with recombinant ADAM17 and purified α5β1 integrin/recombinant β1 integrin in cell free systems:

First, to confirm that ADAM17 is capable of direct integrin binding and to complement our co-immunoprecipitation studies, we employed capture-ELISA. Plates pre-coated with α5β1 integrin antibody were incubated with purified α5β1 integrin, and anti-β1 integrin pre-coated plates were incubated with recombinant β1 integrin. Increasing concentrations of His-tagged recombinant human ADAM17 (0–20 µg/ml) were then added to both plates and the amount of captured ADAM17 was measured. As shown in Figure 3A both purified α5β1 integrin and recombinant β1 integrin were able to bind ADAM17 dose dependently. As negative control BSA-coated surface was used, that showed only negligible ADAM17 binding. These data suggest that not only α5β1 integrin but also β1 integrin alone is capable of ADAM17 binding.

To confirm our data we employed surface plasmon resonance and strong binding was observed between the ADAM17 that was covalently bound to the sensor chip surface and purified α5β1 integrin that was injected over the chip (data not shown).

### Purified α5β1 integrin and recombinant β1 integrin inhibit recombinant ADAM17 activity

After confirming that ADAM17 can directly bind to α5β1 integrin, we sought evidence that direct binding of the integrin regulates ADAM17 enzymatic activity. Recombinant ADAM17 was combined with different molar concentrations of recombinant β1 integrin or with purified, octyl-β-glucoside (OG) extracted α5β1 integrin at a 1∶1 and 1∶10 molar ratio in OG containing buffer. Enzyme activity was measured by addition of a quenched fluorogenic ADAM17 peptide substrate. In this assay, substrate cleavage results in separation of the quencher from the fluorophore, and thus increases the fluorescence intensity of the peptide substrate. Figure 3B shows that both α5β1 integrin and β1 integrin decreased ADAM17 enzymatic activity dose dependently and that maximum inhibition was achieved by 1∶10 molar ratio of integrins to ADAM17.

### β1 integrin regulates HB-EGF shedding by ADAM17 in mesangial cells

**Figure 4:**
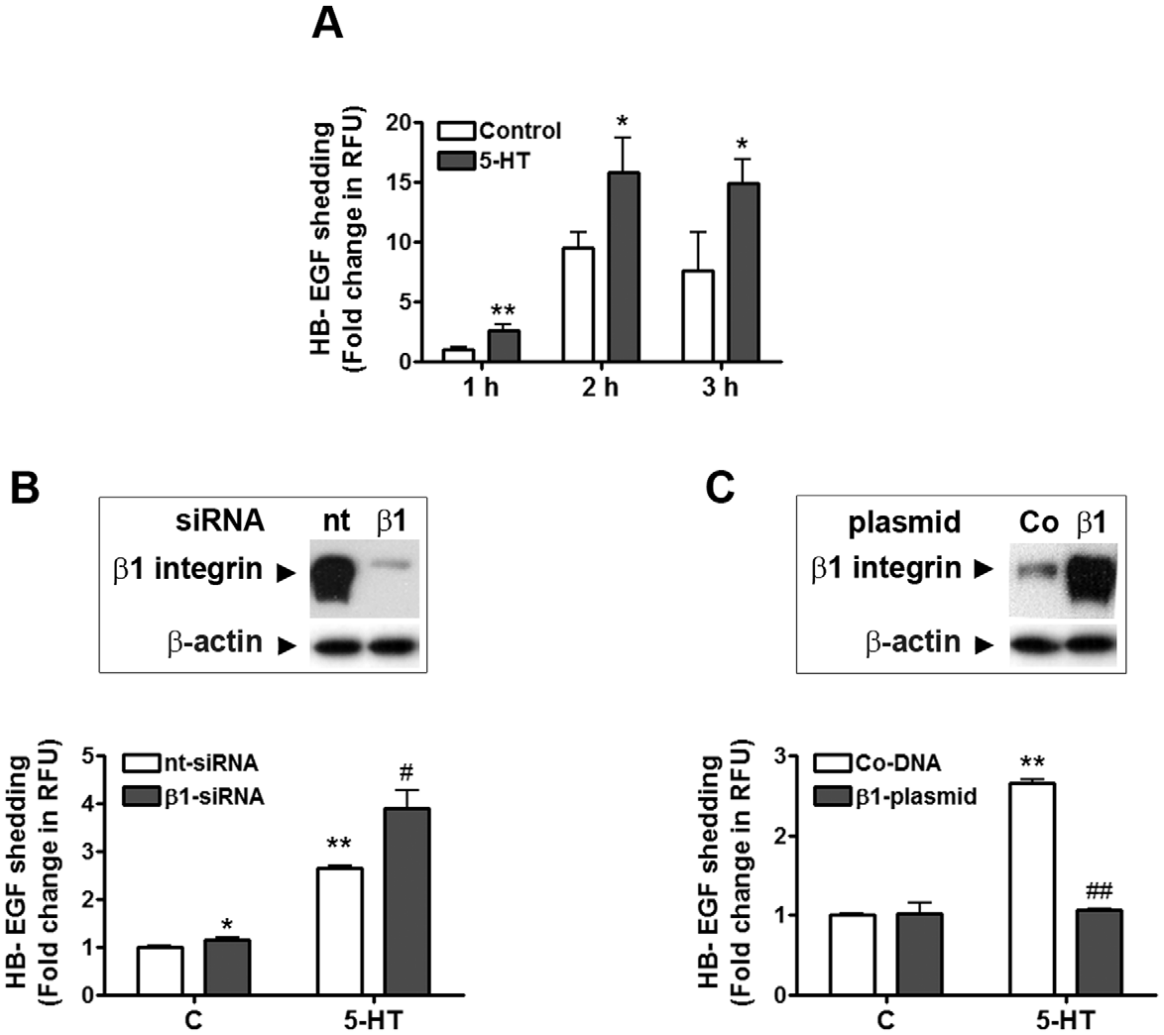
Integrin expression regulates ADAM17 sheddase activity. (A) Time-dependent release of alkaline phosphatase (AP)-tagged HB-EGF by unstimulated (C) and 5-HT stimulated cells. Cells were transfected with AP-HB-EGF expressing plasmid and 2 days after transfection they were stimulated with 5-HT for the indicated time. AP activity of cell supernatants was determined using Attophos substrate. Data are expressed as mean±S.D. of fold change in the rate of change of relative fluorescence units; *p<0.05, **p<0.01 *vs* control at same time point, n = eight experiments, three parallels/each condition. (B) β1 integrin silencing promotes 5-HT-induced AP-HB-EGF shedding. Cells were transfected with AP-HB-EGF expression plasmid together with β1 integrin siRNA (β1-siRNA) or a non-targeting (nt−) siRNA. Successful silencing of β1 integrin was confirmed by resolving the cell lysates on a 4–12% SDS-PAGE and probing for β1 integrin and β-actin (as loading control). Two days after transfection cells were stimulated with 5-HT for 1 h and AP activity of cell supernatants was determined. (C) β1 integrin overexpression inhibits 5-HT induced AP-HB-EGF release in mesangial cells. Cells were transfected with AP-HB-EGF and with β1 integrin expressing plasmid (β1-plasmid) or control DNA (Co-DNA). Successful overexpression of β1 integrin was confirmed by resolving the cell lysates on a 4–12% SDS-PAGE and probing for β1 integrin and β-actin (as loading control). Two days after transfection cells were stimulated with 5-HT for 1 h and AP activity of cell supernatants was determined. Activity data are expressed as mean±S.D. of fold change in the rate of change of relative fluorescence units (RFU); *p<0.05, **p<0.01 *vs* unstimulated control nt-siRNA or control DNA-transfected cells; ^#^ p<0.05 and ^##^ p<0.01 *vs* 5-HT stimulated nt-siRNA or DNA-transfected cells; n = five experiments, three parallels/each condition.

We showed previously that 5-HT stimulation induced ADAM17-dependent HB-EGF shedding in mesangial cells [11]. In the following experiments, we wanted to confirm that β1 integrin regulates ADAM17 sheddase activity. For this purpose, we employed a sensitive method to study ADAM17 activity: we transfected mesangial cells with alkaline phosphatase (AP)-tagged HB-EGF. Two days after transfection, cells were stimulated with 1 µM 5-HT for 1–3 hours as described previously [11], and growth factor release was quantified by measuring the AP activity from the cell conditioned media. We found that 5-HT induced time-dependent increases in AP activity in the medium as an indicator of increased HB-EGF shedding (Figure 4A). With these experiments, we validated AP-HB-EGF as a tool for studying ADAM17 activity in our cell system. As we observed the biggest difference in 5-HT-induced growth factor shedding compared to control at 1 hour (2.6-fold), we chose this timepoint in subsequent experiments.

Since we hypothesized that β1 integrin inhibits ADAM17 activity in mesangial cells we aimed to knock down β1 integrin and study changes in basal and 5-HT-stimulated ADAM17 activity. Mesangial cells were transfected with either (1) AP-HB-EGF construct and a control, non-targeting siRNA (nt-RNA) or (2) AP-HB-EGF construct and β1 integrin silencing RNA (β1-siRNA). Two days after transfection, we stimulated the cells with 5-HT (1 µM) for 1 h and measured AP activity from the cell media as described above. Figure 4B shows that β1 integrin silencing resulted in a moderate, but significant increase in basal ADAM17 activity, and it significantly enhanced 5-HT-induced HB-EGF shedding. The Western blot insert in Figure 4B shows that we achieved almost complete inhibition of β1 integrin expression with our gene silencing approach.

Next, we transfected a β1 integrin-expressing vector into mesangial cells to study whether we can inhibit 5-HT-induced ADAM17 activation. We co-transfected our cells with the following: (1) AP-HB-EGF construct and a control, GFP-expressing vector (Co-DNA) or (2) AP-HB-EGF construct and β1 integrin expression vector (β1-plasmid). Two days after transfection, we stimulated the cells with 5-HT (1 µM) for 1 h and measured AP activity from the cell media. Figure 4C shows that β1 integrin overexpression did not significantly influence basal ADAM17 sheddase activity; however, it completely inhibited 5-HT induced AP-HB-EGF shedding. Successful overexpression of β1 integrin was confirmed by Western blotting (see insert in Figure 4C).

### Integrin activity status regulates ADAM17/α5β1 integrin complex formation

**Figure 5:**
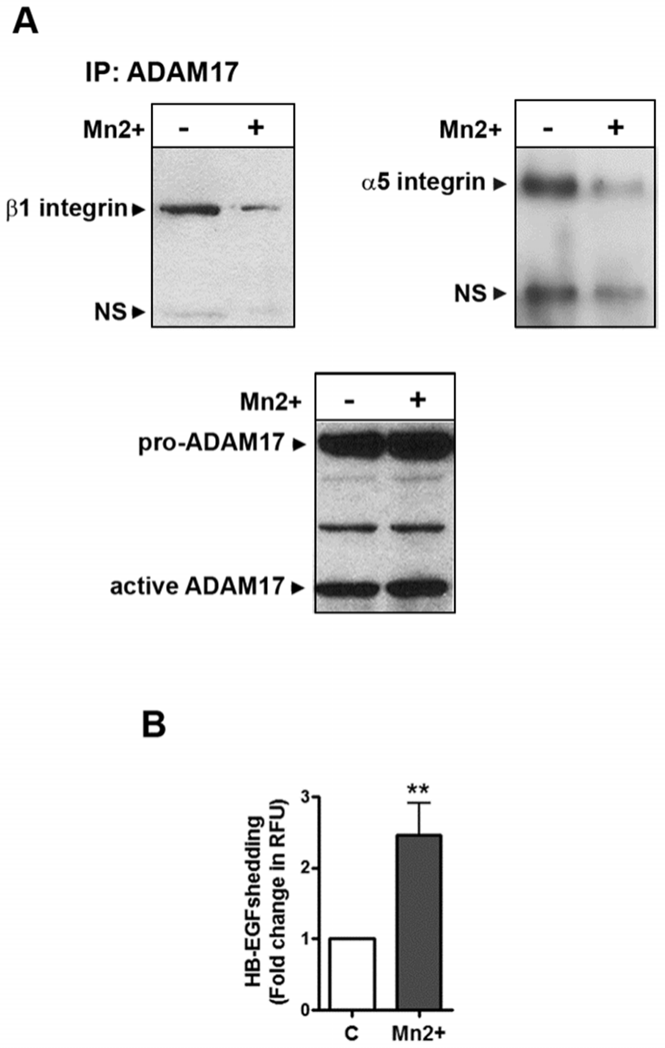
Manganase^2+^ stimulation decreases association of ADAM17 to β1 integrin and leads to increased ADAM17 activity. (A) Control and manganese stimulated (1 mM MnCl_2_ in PIPES buffer for 2 min) mesangial cell lysates were immunoprecipitated with ADAM17 antibody. Samples were resolved on a 3–8% Tris-acetate gel and probed for β1 integrin and α5 integrin by Western blotting. Immunoblotting for ADAM17 served as loading control. Arrows point to specific and non-specific (NS) bands. One representative blot out of three is shown. (B) Mesangial cells were transfected with AP-HB-EGF construct and stimulated with 1 mM MnCl_2_ in PIPES buffer. HB-EGF shedding was expressed as mean±S.D. of fold increase in the rate of change of relative fluorescence units (RFU); **p<0.01 *vs* control; n = three experiments, 6 parallels/each condition.

Conformation of recombinant and purified protein can differ from their three-dimensional in vivo structure. Since in the cell free binding assay we needed 1∶10 molar ratio to achieve a more complete inhibition of ADAM17 activity by the integrins, we hypothesized that conformation of β1 integrin can play an important role in its ADAM17 binding. Therefore, we investigated whether the presence of the divalent cation Mn^2+^, which is known to activate integrin conformation, affects the ADAM17/α5β1 integrin complex formation. Mesangial cells were incubated in control or in 1 mM Mn^2+^ containing medium for 2 minutes then lyzed in OG lysis buffer. Protein complexes were immunoprecipitated with ADAM17 antibody. Immunocomplexes were analyzed in SDS-PAGE followed by Western blotting for α5 and β1 integrin and ADAM17. As shown in Figure 5A, Mn^2+−^-treatment induced a decrease in the ADAM17/α5β1 integrin complex. At the same time, using AP-HB-EGF transfected cells we found that Mn^2+−^-treatment induced a more than 2-fold increase in ADAM17 sheddase activity (Figure 5B). These data suggest that α5β1 integrin conformation indeed regulates integrin binding to ADAM17 and thus the enzyme activity.

## Discussion

All ADAMs have a disintegrin domain, which is potentially capable of integrin binding. However, only a limited number of ADAMs have a catalytically active metalloenzyme domain, which can process membrane bound proteins. This suggests that in catalytically inactive ADAMs integrin-binding may be mainly a cell adhesion-related process as was implicated and explored during the past years [23], [25]. However, we have to hypothesize that in catalytically active ADAMs like ADAM1, −8, −9, −10, −12, −15, −17, −19, −20, −28, −30, −33 integrin binding can also have another role namely, to regulate the activity of the ADAM enzymes. Since the disintegrin domain is in close proximity of the catalytic domain in ADAMs, it is highly possible that protein binding to the disintegrin domain can have significant effect on the enzyme activity by, for example, influencing its substrate binding. Overall, protein-protein interaction can affect the conformation of the binding partners which itself can lead to changes in one or both of the binding partners “activity”. The idea, that integrins can regulate ADAM shedding in vivo was hypothesized by others, too [22]. However, besides data on *in vitro* integrin binding and on interaction between overexpressed integrins and ADAMs, there is still a huge gap in our knowledge about whether integrins indeed bind and regulate ADAM activity *in vivo*.

The immunoprecipitation studies we performed identified α5β1 integrin as the binding partner of ADAM17 in kidney mesangial cells. One of the most important signaling pathways ADAMs participate in is the triple membrane spanning signaling: they mediate crosstalk between GPCRs and receptor tyrosine kinases (RTKs). This mechanism was first described in 1999 [26], and since then its role was extensively studied in various pathophysiological conditions like heart disease [27], cancer [28], and kidney fibrosis [29], [30]. We have shown previously in an *in vitro* model of kidney fibrosis that the GPCR agonist 5-HT induces proliferation through HB-EGF shedding in mesangial cells [11]. Therefore, we investigated the effect of 5-HT on ADAM17/α5β1 integrin assembly. We observed that in mesangial cells GPCR stimulation induced dissociation of the ADAM17/α5β1 integrin complex. This suggested that β1 integrin may have an inhibitory effect on ADAM17 catalytic activity. To our knowledge, this is the first time that integrins were implicated in ADAM17 activation during GPCR stimulation and our subsequent experiments were performed to further confirm this interesting finding.

We used two other detection methods: immunofluorescence co-localization and *in situ* proximity ligation to confirm the close proximity of ADAM17 and α5β1 integrin and to visualize the location of their interaction in primary culture of mesangial cells. Co-immunoprecipitation and co-localization studies can suggest that two molecules are in the same complex however, these methods cannot show with certainty that the proteins directly interact with each other. Therefore, we used the Doulink in situ PLA assay. The great advantage of this method is that (1) it only shows signal if the two antibodies which we employed recognize two different proteins which interact (are in close proximity); (2) because the signal is amplified, it is very sensitive: theoretically each “dot” shows one interaction between two proteins; but more importantly (3) the Duolink assay allowed us to compare ADAM17/α5β1 integrin interactions in control cells to −5-HT treated cells quantitatively. The PLA data confirmed our immunofluorescence findings: in untreated cells ADAM17/α5β1 integrin interaction was localized to the perinuclear area. Further, data analysis showed that compared to control cells after 5-HT treatment only 13% of the ADAM17/α5β1 integrin interaction remained and these were scattered inside the cells, similarly as observed in the confocal immunofluorescence co-localization study.

Our in vitro binding studies using recombinant ADAM17 and β1 integrin and purified α5β1 integrin provided evidence that ADAM17 can bind both α5β1 integrin and β1 integrin directly, without any adaptor protein. Similar finding on α5β1 integrin binding to recombinant (Fc-tagged) ADAM17, purified from COS-1 cells, was previously published [23]. Here we provided evidence that purified recombinant β1 integrin is also capable of ADAM17 binding, and most importantly, we showed that both α5β1 integrin and β1 integrin binding decreased ADAM17 activity. We saw a more complete inhibition of ADAM17 activity when we increased the molar ratio of ADAM17/integrin to 1∶10, which may suggest that the conformation of the purified integrin and recombinant ADAM17 is slightly different from their conformation in the mammalian cellular context. Also, we cannot rule out that other proteins contribute to the ADAM17/integrin binding intracellularly which may make the enzyme inhibition more complete *in vivo*. We explored the importance of integrin conformation in a study in which we used MnCl_2_, a known “integrin activator”. Not only were we able to show that presence of Mn^2+^ causes dissociation of the ADAM17/β1 integrin complex, but at the same time Mn^2+^ increased ADAM17 sheddase activity. These data suggest that α5β1 integrin conformation indeed regulates integrin binding to ADAM17 and the enzyme activity.

In our earlier studies, we determined the sheddase activity of ADAM17 by Western blotting [11], [12]. In order to detect the presence of growth factors from the cell media successfully, we had to concentrate the supernatant of a large number of mesangial cells. To study that β1 integrin regulated ADAM17 sheddase activity in mesangial cells we utilized a more sensitive method. We employed *alkaline phosphatase (AP)-tagged HB-EGF* [31] because we wanted to monitor small enzyme activity changes from a smaller number of cells and we hoped to detect a more immediate response to 5-HT compared to our earlier studies. Using AP-tagged HB-EGF, we were able to repeat our previous observation: 5-HT induced significant increase in shedding of the growth factor. To our disappointment, however, we still needed to stimulate the cells for at least 30 min−1 h with 5-HT in order to see changes in growth factor shedding, even with this sensitive method. We speculated that the reason for this “delayed release” is that some of the growth factors internalize with the EGFR after binding, or perhaps the cleaved growth factors stay bound to cell surface proteoglycans [32]. Therefore, the amount of “released” growth factor can be very small, and a long incubation time is needed in order to detect the shedded growth factor even using this sensitive assay. Nonetheless, we were able to show that 5-HT induced time-dependent release of HB-EGF. Further, our gene silencing and overexpression studies confirmed that β1 integrin is indeed an important negative regulator of ADAM17 activity in kidney mesangial cells during GPCR activation. Interestingly, changes in β1 integrin expression did not affect basal ADAM17 activity as dramatically as we expected. Overexpression of β1 integrin did not significantly decrease the basal activity of the enzyme, but since our immunofluorescence data suggested that most of ADAM17 present in resting mesangial cells are bound to α5β1 integrin, it is highly possible that further increasing the integrin level will have no further effect on ADAM17 activity. The fact, that β1 integrin silencing only moderately increased ADAM17 activity in unstimulated cells is more intriguing. Since ADAM17 is a very important enzyme for the cell survival, it is possible, that besides β1 integrin there are other important regulatory proteins that control the enzyme activity. Removing β1 integrin from the ADAM17 complex can allow rearrangement of regulatory molecules and/or binding of new ones. These possibilities need to be further investigated in the future.

In summary, the data presented in this study provide evidence that binding of α5β1 integrin to ADAM17 has an important role in the activity regulation of the enzyme. Further, to our knowledge this is the first paper to report that GPCR engagement leads to ADAM17 activation through dissociation of an integrin from the enzyme. This novel observation is an important step in our understanding in how ADAM17 (or potentially other catalytically active ADAMs) can be regulated by integrins. Further, since there is increasing evidence for the pathophysiological role of ADAM17 in CKD and other metabolic and inflammatory diseases, we suggest that targeting the integrin-binding of the enzyme can be used to develop novel therapies for these disorders in the future.

## Materials and Methods

### Cell Culture

#### Ethics Statement

Primary rat mesangial cells were obtained from cortical section of kidneys from 100–150 gram Sprague-Dawley rats by collagenase treatment and a standard sieving technique as described previously [33]. The kidneys were harvested in accordance with the protocol specifically approved for this study by the Institutional Animal Care and Use Committees of the Medical University of South Carolina.

Cells were cultured in RPMI-1640 medium supplemented with 20% (v/v) fetal bovine serum (FBS) and antibiotics (100 units/ml penicillin and 100 µg/ml streptomycin) at 37°C in a humidified atmosphere of 95% air and 5% CO_2_. Cells were subcultured weekly and used between passages 6–14. All cell culture reagents were purchased from Invitrogen (Carlsbad, CA).

### Co-Immunoprecipitation and Western Blotting

Mesangial cells were seeded into culture plates, grown to 80% confluency and serum starved for 2 days in 0.5% (w/v) bovine serum albumin (BSA)-containing medium. Cells were then treated with vehicle or with 1 µM of 5-HT for 10 min. Cell were lysed after stimulation in 1% Triton lysis buffer (Boston Bioproducts, Ashland, MA) containing protease inhibitors (Protease inhibitor cocktail set III, EMD Biosciences, San Diego, CA) and phosphatase inhibitors (Halt™ Phosphatase Inhibitor Cocktail, Thermo Scientific, Rockford IL) on a rotating shaker at 4°C for 30 min. Equal protein amount (∼1 mg) of cell lysates were incubated overnight at 4°C with ADAM17-specific antibody (MAB2129, R&D Systems, Minneapolis, MN). After incubation at 4°C for 1 h with protein G-agarose beads (Santa Cruz Biotech, Santa Cruz, CA), immunoprecipitated protein complexes were washed with lysis buffer and eluted with SDS-PAGE sample buffer. Protein complexes were resolved by SDS-PAGE using either 3–8% Tris-acetate gel or 4–12% Bis-Tris gel (Invitrogen, Carlsbad, CA) as described before [34] and analyzed by immunoblotting using 1∶100 dilution of α5 integrin antibody (sc-10729, Santa Cruz Biotechnology, Santa Cruz, CA) or 1∶500 dilution of β1 integrin-specific antibody (sc-8978, Santa Cruz Biotechnology, Santa Cruz, CA). Blots were then re-probed using 1∶5,000 dilution of ADAM17-antibody (MAB2129, R&D Systems, Minneapolis, MN). Immunoprecipitations with non-immune IgG or protein G-agarose beads alone served as negative controls.

### Immunofluorescence staining and Confocal Microscopy

Cells were grown on 35 mm lysine-coated glass-bottom culture dishes (MatTek Corporation, Ashland, MA). After treatments with 1 µM of 5-HT, cells were fixed with freshly prepared 2% paraformaldehyde in PBS containing 0.2% Triton X-100 for 15 min at room temperature. Nonspecific binding sites were blocked with 1% bovine serum albumin in PBS for 1 h. Cells were incubated with 1∶100 dilution (2 µg/ml) of ADAM17-specific antibody (sc-6416, Santa Cruz, Santa Cruz, CA) and with 1∶50 dilution of α5β1 integrin antibody (MAB1969, Millipore, Billerica, MA). Negative controls were incubated only with buffer. Incubations with 1∶500 dilutions of Alexa Fluor-conjugated appropriate secondary antibodies (Invitrogen, Carlsbad, CA) were performed in blocking solution. Confocal microscopy was performed using a Leica laser-scanning microscope (Wetzlar, Germany).

### In situ proximity ligation assay (PLA)

We used the Doulink in situ PLA reagent from Olink Biosciences (Uppsala, Sweden) to characterize endogenous protein interactions according to the manufacturer's recommendation. Cells were grown on glass bottom culture dishes then treated and handled as for immunofluorescence staining above. After incubating with ADAM17 and α5β1 integrin antibodies for 1 h at room temperature oligo-labelled anti-goat plus and anti-mouse minus PLA probes were used as recommended by the manufacturer. Negative control slides were incubated either with ADAM17 antibody or with α5β1 integrin antibody only before incubation with PLA probes. Samples were mounted with the Duolink mounting medium. PLA images (fluorescence dots) were acquired using a Leica laser-scanning microscope (Wetzlar, Germany) and analyzed by “BlobFinder”, a freeware developed by the Centre for Image Analysis, Uppsala University, Sweden (http://www.cb.uu.se/~amin/BlobFinder).

### In Vitro Binding Assay

Direct binding of ADAM17 and integrins was analyzed using capture-ELISA assay. ELISA plates were coated with 5 mg/ml of α5β1 integrin antibody (Millipore, MAB1969), or with β1 integrin antibody (Santa Cruz, sc-8978) in 50 mM Na_2_CO_3_ buffer (pH 9.6) at 4°C overnight. Plates were then blocked with 5% (w/v) RIA grade BSA (Sigma-Aldrich, St. Louis, MO), then incubated with recombinant human β1 integrin (H00003688-P01, Abnova, Taipei City, Taiwan) or with purified α5β1 integrin (Millipore, Billerica, MA) in Tris buffer (pH 7.4) containing 25 mM octyl-D-gluco-pyranoside, 0.5 mM CaCl_2_, 1 mM MgCl_2_ and 0.5 mM MnSO_4_ overnight at 4°C. The next day, plates were washed and recombinant human ADAM17 (cat#930-ADB, R&D Systems, Minneapolis, MN), was added at concentrations indicated. After 3 h of incubation at room temperature, His-probe horseradish peroxidase (Thermo Scientific, Waltham, MA) was added as suggested by the manufacturer for 15 min at room temperature. Peroxidase activity as a measure of bound ADAM17 was determined using 1 step ultra TMB (3,3′,5,5′-tetramethylbenzidine) chromogenic substrate according to the manufacturer's recommendation (Thermo Scientific, Waltham, MA). Reaction was terminated with 2 N sulfuric acid and absorbance was read at 450 nm by a SpectraMax M5 fluorescence plate reader (Molecular Devices, Sunnyvale, CA).

### Fluorescence Enzyme Activity Assay

Recombinant ADAM17 was purchased from R&D Systems (Minneapolis, MN) and purified, octyl-β-glucoside (OG) extracted α5β1 integrin was from Millipore (Billerica, MA). Recombinant ADAM17 (10 ng/well) was measured alone or ADAM17 was combined with α5β1 integrin at a 1∶1 and 1∶10 molar ratio in OG containing buffer (50 mM Tris, 150 mM NaCl, 25 mM OG, 1 mM CaCl_2_, 1 mM MgCl_2_ and 0.5 mM ZnSO_4_) in 96 well plates. The quenched fluorogenic ADAM17 substrate MCA-Pro-Leu-Ala-Gln-Ala-Val-Dpa-Arg-Ser-Ser-Ser-Arg-NH_2_ (EMD Biosciences, San Diego, CA) was then added at a 6 µM final concentration, and enzyme activity was measured as the rate of change of relative fluorescence units (ΔRFUs^−1^) using a Spectramax M5 spectrofluorometer (Molecular Devices, Sunnyvale, CA) with excitation at 320 nm and emission at 405 nm.

### Alkaline Phosphatase (AP)-HB-EGF Shedding Assay

Mesangial cells were seeded into 12-well tissue culture plates and transfected with alkaline phosphatase (AP)-tagged HB-EGF expressing construct alone or in combination with either β1 integrin-expressing vector (Genecopoeia, Rockville, MD) or with β1 integrin siRNA (Dharmacon, Lafayette, CO) using Dharmafect Duo reagent (Dharmacon, Lafayette, CO) as directed by the manufacturer. As negative controls, we used GFP-expressing vector and non-targeting siRNA, respectively. Two days after transfection the cells were stimulated with 1 µM 5-HT for the indicated length of time, and growth factor release was quantified by measuring the AP activity of the medium using a sensitive alkaline phosphatase substrate (Attophos, Roche, Indianapolis, IN) and the SpectraMax M5 fluorescence plate reader (Molecular Devices, Sunnyvale, CA).

### Integrin conformation studies

For integrin conformation studies cells were incubated in 1 mM MnCl_2_-containing PIPES buffer (50 mM pH 7.4) for 2 minutes. Cells were then lyzed in OG lysis buffer (50 mM Tris pH 7.4, 25 mM octyl-D-glucopyranoside and 150 mM NaCl) containing protease inhibitors (Protease inhibitor cocktail set III, EMD Biosciences, San Diego, CA). ADAM17/integrin complexes were immunoprecipitated using ADAM17 antibody and analyzed in SDS-PAGE followed by ADAM17 and β1 integrin Western blotting as described above. For shedding experiments, mesangial cells were seeded into 12 well plates and transfected with the AP-HB-EGF. The cells were washed with PBS and incubated in 50 mM PIPES buffer (pH 7.4) with or without 1 mM Mn^2+^ for 1 h and HB-EGF shedding was measured as described above.

### Statistical Analysis

For data analysis, Student's t-test and ANOVA followed by Newmann-Keul post hoc comparison were performed using Prism Statistics Software (GraphPad, La Jolla, CA). *P* values <0.05 were regarded as statistically significant.
